# Unilateral Upper Extremity Paralysis Secondary to Hypokalemia and Fasting: A Case Report

**DOI:** 10.5811/cpcem.47252

**Published:** 2025-09-15

**Authors:** Alexander Adler, Samy Shelbaya, Sean McCormick

**Affiliations:** * Wayne State University, Department of Emergency Medicine, Detroit, Michigan; † Wayne State University, School of Medicine, Detroit, Michigan

**Keywords:** *case report*, *hypokalemia*, *paralysis*, *unilateral*

## Abstract

**Introduction:**

Paralysis from hypokalemia commonly presents with generalized weakness; however, in rare cases it may present with unilateral or focal symptoms. Unilateral paralysis in hypokalemia is particularly challenging due to its mimicry of central nervous system (CNS) disorders such as ischemic stroke. Patients often undergo extensive and costly neuroimaging before a metabolic etiology is recognized.

**Case Report:**

A 19-year-old male presented to the emergency department reporting an abrupt onset of inability to hold things in his right hand. He denied any precipitating factors but did note that he was fasting for the Muslim holy month of Ramadan. On exam, the patient was seen to have absent grip strength in the right hand. The patient’s metabolic panel showed hypokalemia with a potassium of 2.4 millimoles per liter (mmol/L) (reference range: 3.5 to 5.2 mmol/L). Following neurology consultation, we determined that the patient’s focal weakness was secondary to hypokalemia, possibly triggered by his fasting. The patient was given potassium chloride 120 milliequivalents by mouth, and repeat potassium had increased to 3.2 mmol/L. The patient was re-evaluated and reported that his symptoms had completely resolved.

**Conclusion:**

Cases of focal weakness due to hypokalemia can occur. Primary CNS causes should be ruled out prior to making the diagnosis. Treatment should be focused on potassium repletion and avoidance of triggers. If hypokalemic periodic paralysis is a concern, neurology follow-up should be arranged for definitive diagnosis with electromyography.

## INTRODUCTION

Hypokalemia, defined as a serum potassium level below 3.5 millimoles per liter (mmol/L), is a frequent electrolyte disturbance in clinical practice with diverse etiologies and clinical manifestations. Hypokalemia-induced paralysis is attributed to hyperpolarization of skeletal muscle cell membranes, rendering the muscle fibers electrically unexcitable and leading to failure of conduction of action potentials. Common presentations include generalized muscle weakness and arrhythmias. However, hypokalemia may present as unilateral paralysis posing unique diagnostic challenges. Instances of unilateral paralysis secondary to hypokalemia are rare. Physiologically, asymmetric or focal presentations of hypokalemic periodic paralysis may involve subclinical corticospinal pathway damage or pre-existing structural abnormalities unmasked by potassium depletion.^1^

The etiologies of hypokalemia include renal losses due to distal renal tubular acidosis and primary hyperaldosteronism, gastrointestinal losses due to diarrhea or vomiting, and intracellular shifts caused by insulin administration or beta-adrenergic activity. Unilateral paralysis in hypokalemia is particularly challenging due to its mimicry of central nervous system (CNS) disorders such as ischemic stroke.^2^ This emphasizes the need for timely neural electrophysiological examinations and potassium supplementation if an initial CNS etiology is not discovered.^3^ Furthermore, malnutrition-induced hypokalemia can result in focal neurological deficits, underscoring the importance of nutritional history in diagnostic workups.^4^ Assessing a patient with unilateral symptoms requires a high index of suspicion for accurate diagnosis and timely management.

## CASE REPORT

A 19-year-old right-hand dominant male presented to the emergency department (ED) during an overnight shift complaining of right-hand weakness that started approximately 12 hours prior to presentation. He denied any significant past medical history or previous episodes. He reported an abrupt onset of symptoms, finding it difficult to hold things in his right hand. He denied any precipitating factors but did note that he was fasting for the Muslim holy month of Ramadan. His symptoms seemed to be localized to the right hand only. He denied any slurred speech, facial weakness, or involvement of his other extremities.

At triage, the patient’s blood pressure was 106/62 millimeters of mercury, heart rate 85 beats per minute, respiration rate 18 breaths per minute, temperature 36.7°C, and pulse oximetry was 97% on room air. On physical examination, the patient was well appearing, in no acute distress. His cranial nerves were intact. He had full sensory and motor function of the left upper extremity and lower extremities bilaterally. However, the patient had absent grip strength (0/5) in the right hand. The patient appeared to have full strength in the right wrist, elbow and remainder of the right upper extremity.

Considering the patient’s sudden-onset, unilateral, focal neurological symptoms, neurology was consulted. Given the patient’s current religious fasting, there was concern for an electrolyte disturbance. The patient’s complete blood count showed mild anemia with a hemoglobin of 12.1 grams per deciliter (g/dL) (reference range: 13.6–17.2 g/dL) but otherwise was within normal limits. The patient’s complete metabolic panel showed hypokalemia with a potassium of 2.4 millimoles per liter (mmol/L) (3.5–5.2 mmol/L), hypocalcemia with a calcium of 7.1 milligrams per deciliter (mg/dL) (8.5–10.2 mg/dL), and hypomagnesemia with a magnesium of 1.4 mg/dL (1.6–2.2 mg/dL). With neurology’s assistance we diagnosed the patient with muscle weakness secondary to hypokalemia. Later, additional history was obtained that the patient had an episode of generalized weakness four months earlier that had resolved spontaneously. There was concern the patient may also have had hypokalemic periodic paralysis that was triggered by his current fasting.

Neurology did recommend potassium repletion to a level greater than 4 mmol/L and re-evaluation. The patient was given potassium chloride 40 milliequivalents (mEq) by mouth. The patient was given three doses for a total of 120 mEq of potassium. Repeat laboratory studies were sent, and repeat potassium had increased to 3.2 mmol/L.

*CPC-EM Capsule*

What do we already know about this clinical entity?

*Hypokalemia can present as a central nervous system disorder mimic, usually with symptoms of generalized weakness*.

What makes this presentation of disease reportable?

*This is the first known case of focal weakness secondary to hypokalemia due to religious fasting followed by a large meal*.

What is the major learning point?

*In some instances, hypokalemia-induced paralysis may present with unilateral symptoms and should be considered in the differential diagnosis for acute stroke*.

How might this improve emergency medicine practice?

*A thorough dietary and nutritional history may aid in the timely diagnosis and treatment of electrolyte disturbances as the cause of muscle weakness*

The patient was re-evaluated. He reported that his symptoms had completely resolved. Despite being informed of the neurology team recommendation to replete potassium to a level ≥ 4 mmol/L, at this point the patient declined further electrolyte replacement from the ED. He reported that since it was almost sunrise, his window to eat was closing and that he needed to leave the ED or he would not be able to eat again until sunset. Therefore, the patient was discharged.

## DISCUSSION

This case represents a rare manifestation of hypokalemia resulting in focal weakness. Typically, most cases of hypokalemic periodic paralysis result in paralysis that symmetrically affects skeletal muscle cells in those patients with the requisite mutations to be susceptible to this condition. These involve mutations in CACNA1S (a voltage-gated calcium channel found in the transverse tubules of skeletal muscle cells), SCN4A (a voltage-gated sodium channel found in the neuromuscular junction), and KCNJ2 (an inward rectifier potassium channel).^5^ These mutations are loss of function mutations, impeding the affected channel’s ability to function normally. In patients with mutations in CACNA1S and SCN4A, the outcome involves reduced excitability and a reduced ability to depolarize the skeletal muscle cell. This interferes with its ability to contract, resulting in paralysis. Mutations in KCNJ2 will result in an inability to repolarize the skeletal muscle cell, resulting in a similar clinical manifestation and the additional association of cardiac arrhythmias resulting in Andersen-Tawil syndrome. This is a form of long QT syndrome resulting in a prolonged QT interval, ventricular ectopy, and ventricular tachycardia.^6^

These mutations precipitate muscle weakness due to hypokalemia in several ways. First, low extracellular potassium concentration will cause the skeletal muscle cell to repolarize to its resting potential more quickly. This is due to an increased chemical gradient between the intracellular concentration of potassium and the extracellular concentration of potassium. This results in less sustained depolarization, which makes it more difficult for the skeletal muscle cell to reach the threshold by which it can contract. Additionally, it also results in premature muscle relaxation.^7^ In patients with hypokalemic periodic paralysis, an existing channelopathy in CACNA1S and SCN4A will exacerbate the normal effect of hypokalemia, resulting in insufficient depolarization to the threshold potential to initiate muscle contraction. In patients with mutations in KCNJ2, there is impeded repolarization back to the resting potential, resulting in a decreased electrical gradient for depolarization. This leads to a less excitable membrane and less forceful muscle contraction.^8^

In our patient, the most likely factor for his symptoms was his dietary habits during Ramadan. An undiagnosed mutation in one of the previously described channels could have also been a factor. During Ramadan, people who observe the Muslim holy month will fast from dawn to sunset and can eat between sunset and dawn. This patient presented to the ED several hours after sunset, after eating a large meal following a prolonged period of fasting. This sort of dietary pattern will often result in hypokalemia due to significant amounts of insulin being secreted in response to the large increase in blood glucose. Insulin is thought to decrease extracellular potassium concentration by increasing the translocation of the sodium-potassium pump to the surface of skeletal muscle cells.^9^ This patient possibly also had one of the above mutations, which resulted in the clinical manifestation of right upper-extremity focal weakness.

Although most cases of hypokalemic periodic paralysis present with generalized paralysis, case reports of focal paralysis have also been discussed in the literature. Ma et al describes eight patients with focal paralysis in the setting of hypokalemia, which subsequently resolved following potassium repletion as was the case in the patient described here.^3^ Negrotto et al describe case reports of unilateral weakness due to Sjogren disease and hyperaldosteronism. Further triggers of stroke-like symptoms have been reported from anti-hypertensive use and a nutrient-poor diet of primarily ramen noodles.^2^,^4^ The underlying mechanism resulting in focal paralysis as opposed to generalized paralysis is not completely understood. Some studies indicate that sodium-potassium pump activity may be asymmetrically distributed in skeletal muscle cells in response to corticospinal tract involvement.^10^ It should also be noted that patients with focal paralysis are at risk for developing generalized paralysis; thus, prompt diagnosis and management is necessary to prevent further complications.^2^

This case illustrates the importance of including hypokalemia on the differential diagnosis for unilateral focal weakness, especially in the setting of common triggers such as high carbohydrate meals, meals with high sodium content, rest after strenuous exercise, and sudden changes in temperature. Thus, a thorough history must be taken; in addition, other etiologies involving the CNS should be ruled out. Although the condition can be diagnosed clinically, the gold standard for diagnostic testing is through an electromyographic exercise test.^11^ Patients diagnosed with this condition clinically should be referred to a neurologist for outpatient follow-up and so that electromyographic testing can be conducted to confirm the diagnosis.

Treatment of the condition involves resolving acute symptoms and preventing further attacks. The importance of diagnosing the disorder early is paramount since future attacks can be more severe and potentially life-threatening, since they have the potential to cause cardiac arrhythmias and respiratory depression. Potassium repletion is generally performed orally, unless the patient is unable to tolerate oral intake. Repletion with 10 mEq of potassium is typically expected to raise serum potassium by 0.1 mEq/L immediately after administration. Daily potassium supplementation is often required, with 100–150 mEq of potassium often needed to manage daily fluctuations in muscle strength and function.^11^ Additionally, the patient should be informed of common triggers and how to avoid them. Acetazolamide and spironolactone can also be prescribed to prevent future attacks.^12^ If there is strong family history, disorders can be diagnosed using gene-targeted testing (multigene panel), whereas those in whom the diagnosis of hypokalemic periodic paralysis has not been considered are more likely to be diagnosed using genomic testing (exome sequencing and genome sequencing).^13^

## CONCLUSION

In patients who are susceptible to hypokalemia, cases of focal weakness can occur and should be considered in the differential diagnosis. Primary central nervous system causes should be ruled out prior to making the diagnosis. Treatment should be focused on potassium repletion to resolve the acute symptoms, and preventative measures should be taken to avoid triggers for future attacks. Outpatient follow-up with neurology should be arranged prior to discharge so that the disorder can be definitively diagnosed with electromyographic testing. Genetic testing for ion channel mutations should also be considered in patients with a strong family history of concerning symptoms.
